# *Now you see me*: lights on Merkel Cell Carcinoma

**DOI:** 10.1186/s41824-023-00164-7

**Published:** 2023-03-20

**Authors:** Cristiano Pini, Giovanni Matassa, Fabrizia Gelardi, Lidija Antunovic

**Affiliations:** 1 Department of Biomedical Sciences, Humanitas University, Via Rita Levi Montalcini 4, 20090 Pieve Emanuele, Milan, Italy; 2 IRCCS Humanitas Research Hospital, Via Manzoni 56, 20089 Rozzano, Milan, Italy; 3 IRCCS Policlinico San Matteo di Pavia, Viale Camillo Golgi 19, 27100 Pavia, Italy

## Abstract

Merkel Cell Carcinoma (MCC) is a rare primary cutaneous cancer with aggressive behaviour and poor prognosis. Although MCC cells express somatostatin receptors (SSTR), SSTR-targeted PET/CT is not routinely performed in clinical practice. In contrast, the use of [^18^F]FDG PET/CT is more widespread and its prognostic role is well established. We present the case of an MCC patient suspected recurrence who underwent restaging with both [^18^F]FDG and [^68^ Ga]Ga-DOTA-TOC PET/CT. [^18^F]FDG PET/CT showed pathological uptake only in mediastinal lymph nodes, but SSTR imaging also revealed multiple liver and skeletal metastases, leading to significant disease upstaging and relevant changes in the therapeutic management.

## Introduction

Merkel Cell Carcinoma (MCC) is a rare primary skin malignancy, accounting for less than 1% of all skin cancers. MCC is an aggressive tumour, with five-year overall survival rates ranging from 48 to 63% (Gauci et al. [Bibr CR7][Bibr CR8]). Imaging with Positron Emission Tomography/Computed Tomography (PET/CT) is generally not recommended at baseline for early-stage MCC, although it is widely used for locally advanced and metastatic disease (Gauci et al. 2022a). MCC cells present a neuroendocrine differentiation and express somatostatin receptors (SSTR), providing a potential theranostic target (Gardair et al. 2015). However, PET/CT targeting SSTR is not routinely performed in clinical practice (Gauci et al. 2022b). Conversely, [^18^F]Fluorodeoxyglucose (FDG) PET/CT showed a higher sensitivity compared to CT in detecting hypermetabolic distant metastases at staging in patients with advanced disease (Hawryluk et al. 2013). In addition, [^18^F]FDG PET/CT provides relevant prognostic information in restaging and post-treatment settings (Byrne et al. 2015; Taralli et al. 2018).

## Case presentation

An 85-year-old patient was diagnosed with MCC in the right temporal region without lymph node involvement and underwent surgical excision of the primary skin lesion. One year later, the patient had a local skin recurrence, with localisations in the ipsilateral laterocervical lymph nodes and parotid gland. Consequently, the patient was treated with right temporal skin radicalisation, completion ipsilateral lymph node dissection and right parotidectomy. Within one year, a CT scan revealed the presence of four liver metastases, and the patient subsequently underwent chemotherapy with carboplatin and etoposide, achieving a complete pathological response on [^18^F]FDG PET/CT. Follow-up was negative for four years. Afterwards, the disease relapsed in the middle pulmonary lobe and the patient was referred to our hospital for stereotactic radiotherapy. Six months later, suspicious mediastinal lymph nodes appeared on CT scan, along with findings related to post-actinic changes in the middle pulmonary lobe.

**Fig. 1:**
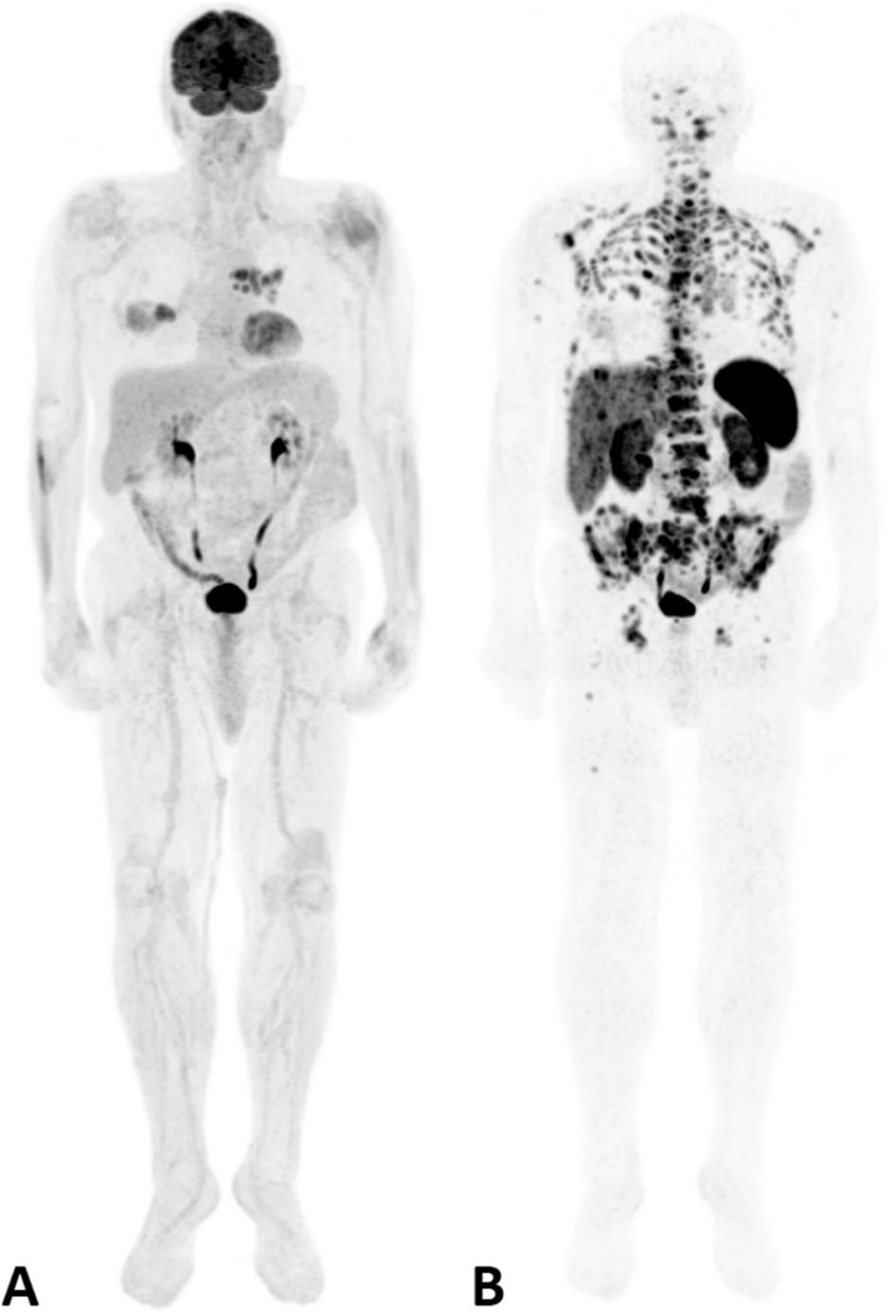
Maximum Intensity Projection (MIP) images of [^18^F]FDG PET/CT (**a**) and [^68^Ga]Ga-DOTA-TOC PET/CT (**b**)

**Fig. 2:**
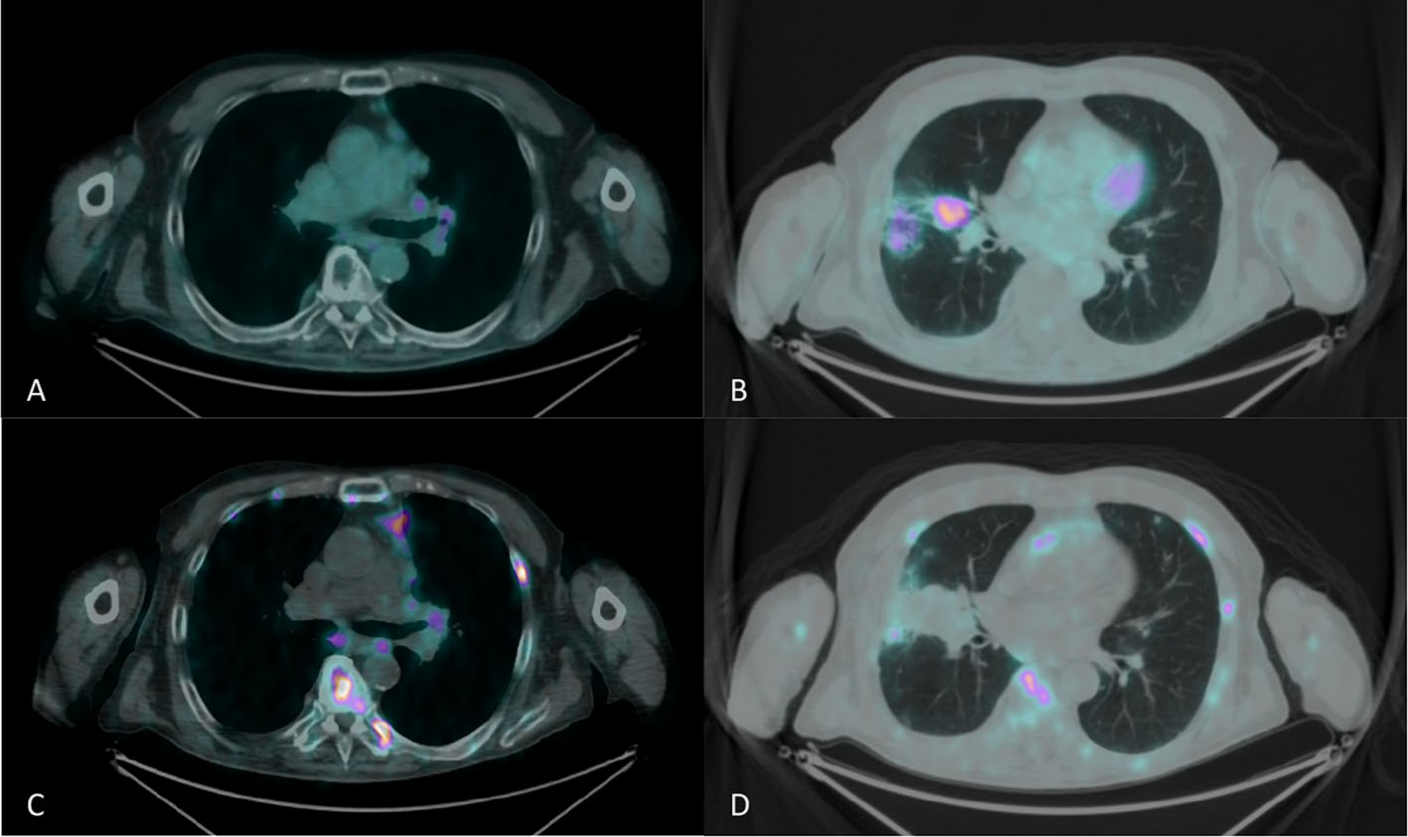
[^18^F]FDG PET/CT showed increased uptake in mediastinal lymph nodes (**a**) and post-actinic changes in the middle pulmonary lobe (**b**). [^68^Ga]Ga-DOTA-TOC PET/CT confirmed pathological mediastinal lymph nodes (**c**), while showing no significant uptake in the post-actinic alterations of the middle lobe (**d**)

**Fig. 3:**
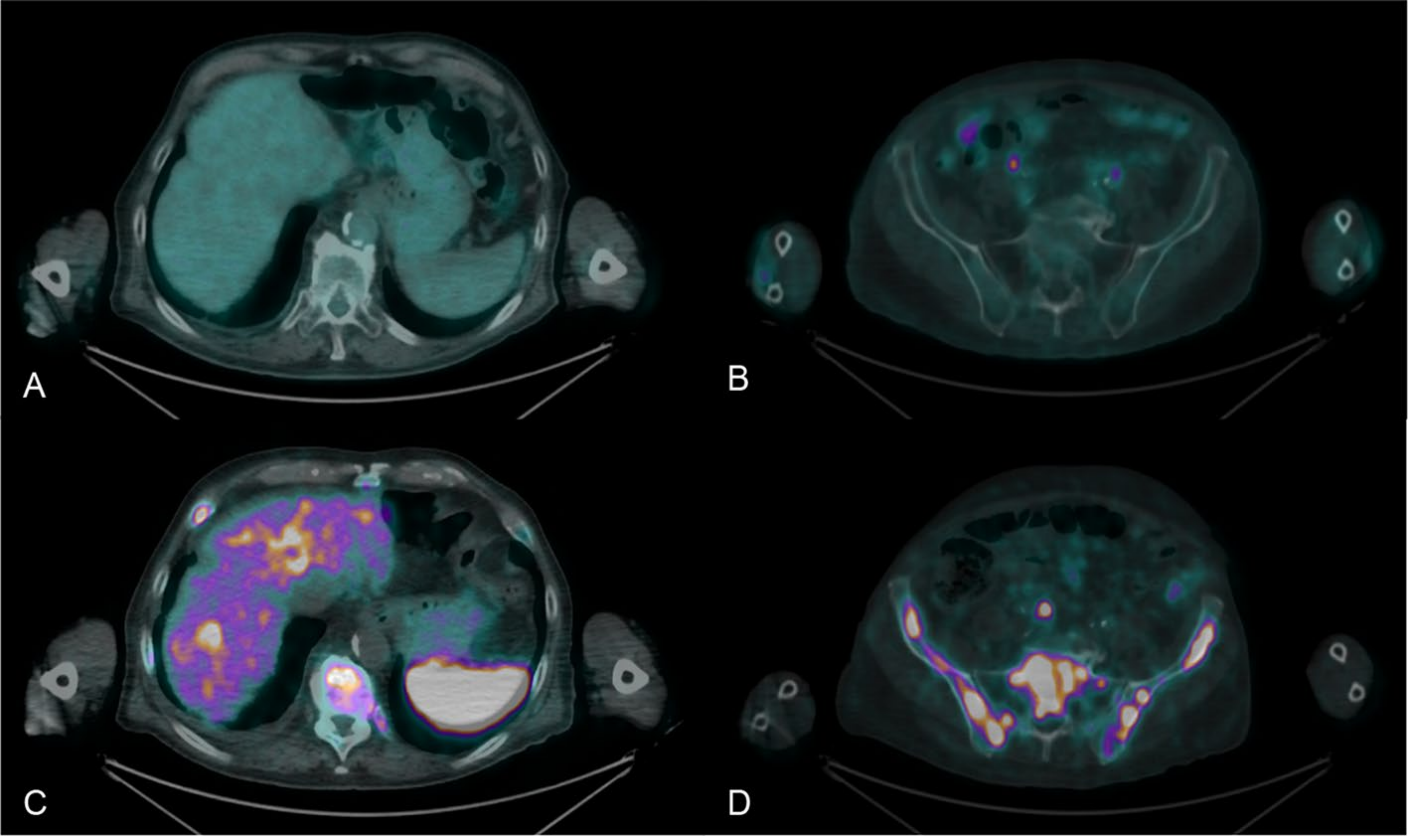
[^18^F]FDG PET/CT showed no significant uptake both in the liver parenchyma (**a**) or skeletal district (**b**). In contrast, [^68^Ga]Ga-DOTA-TOC PET/CT revealed pathologically inhomogeneous uptake in the liver parenchyma (**c**) and multiple lesions with increased uptake in the pelvic bones (**d**)

The disease was restaged with both [^18^F]FDG and [^68^ Ga]Ga-DOTA-TOC PET/CT within less than three months (Fig. 1). [^18^F]FDG PET/CT showed increased uptake in the mediastinal lymph nodes (Fig. 2a), together with inhomogeneous uptake related to radiation-induced changes in the middle pulmonary lobe (Fig. 2b). Besides confirming the pathological mediastinal lymph nodes (Fig. 2c), [^68^Ga]Ga-DOTA-TOC PET/CT revealed multiple liver metastases (Fig. 3c) and extensive skeletal involvement (Fig. 3d). The middle lobe lung alterations showed no significant tracer uptake (Fig. 2d), confirming their post-actinic nature.

[^68^ Ga]Ga-DOTA-TOC PET/CT resulted in significant disease upstaging in as much as it revealed pathological lesions in the liver parenchyma and skeleton that did not show significant uptake on [^18^F]FDG PET/CT (Fig. 3a, b, respectively). Management of the patient was therefore changed from a local approach with radiotherapy alone to systemic treatment.

Although not routinely performed in MCC patients, SSTR PET/CT demonstrates a high accuracy in detecting bone, soft tissue, and brain metastases, highlighting the burden of well-differentiated disease (Buder et al. 2014; Sollini et al. 2016). Furthermore, SSTR PET/CT paves the way for innovative therapeutic approaches exploiting SSTR, including somatostatin analogues and peptide receptor radionuclide therapy with [^177^Lu]Lu-DOTATATE (Akaike et al. 2021; Askari et al. 2022).

SSTR expression in MCC is highly heterogeneous and, in contrast to other neuroendocrine tumours, does not correlate with disease severity, either in terms of clinical features, Ki67 proliferative index or clinical outcome (Gardair et al. 2015). On the other hand, a high FDG avidity is a non-specific marker of increased metabolic activity in several malignancies. [^18^F]FDG shows the same biological behaviour in MCC, reflecting its aggressiveness (Concannon et al. 2010). Only a few studies compared the performance of [^18^F]FDG and SSTR PET/CT in the assessment of MCC, showing almost comparable results (Taralli et al. 2018; Epstude et al. 2013).

However, the use of a single radiopharmaceutical would only provide a partial outlook of the disease, given its high heterogeneity and aggressiveness. This case report supports a dual-tracer approach in patients with advanced and recurrent MCC for a more accurate assessment of the disease burden.

## Conclusion

This case emphasises the potential clinical impact of SSTR PET/CT and its ability, in combination with [^18^F]FDG PET/CT, to shed light on the complex biology of MCC.

## Data Availability

The manuscript represents valid work. Lidija Antunovic had full access to all the data and takes responsibility for the data integrity and the accuracy of the data analysis.

## References

[CR1] Akaike T, Qazi J, Anderson A, Behnia FS, Shinohara MM, Akaike G (2021). High somatostatin receptor expression and efficacy of somatostatin analogues in patients with metastatic Merkel cell carcinoma. Br J Dermatol.

[CR2] Askari E, Moghadam SZ, Wild D, Delpassand E, Baldari S, Nilica B, et al. Peptide receptor radionuclide therapy in Merkel cell carcinoma: a comprehensive review. J Nucl Med Technol; 2022. 10.2967/jnmt.122.264904 36195446

[CR3] Buder K, Lapa C, Kreissl MC, Schirbel A, Herrmann K, Schnack A (2014). Somatostatin receptor expression in Merkel cell carcinoma as target for molecular imaging. BMC Cancer.

[CR4] Byrne K, Siva S, Chait L, Callahan J, Bressel M, Seel M (2015). 15-year experience of 18F-FDG PET imaging in response assessment and restaging after definitive treatment of Merkel cell carcinoma. J Nucl Med.

[CR5] Concannon R, Med B, Larcos GS, Veness M. The impact of 18 F-FDG PET-CT scanning for staging and management of Merkel cell carcinoma: results from Westmead Hospital, Sydney, Australia. 2010. 10.1016/j.jaad.2009.06.021 20082888

[CR6] Epstude M, Tornquist K, Riklin C, di Lenardo F, Winterhalder R, Hug U (2013). Comparison of (18)F-FDG PET/CT and (68)Ga-DOTATATE PET/CT imaging in metastasized Merkel cell carcinoma. Clin Nucl Med.

[CR7] Gardair C, Samimi M, Touzé A, Coursaget P, Lorette G, Caille A (2015). Somatostatin receptors 2A and 5 are expressed in merkel cell carcinoma with no association with disease severity. Neuroendocrinology.

[CR8] Gauci ML, Aristei C, Becker JC, Blom A, Bataille V, Dreno B (2022). Diagnosis and treatment of Merkel cell carcinoma: European consensus-based interdisciplinary guideline—update 2022. Eur J Cancer Pergamon.

[CR9] Gauci M-L, Aristei C, Becker JC, Blom A, Bataille V, Dreno B (2022). Diagnosis and treatment of Merkel cell carcinoma: European consensus-based interdisciplinary guideline: update 2022. Eur J Cancer.

[CR10] Hawryluk EB, O’Regan KN, Sheehy N, Guo Y, Dorosario A, Sakellis CG (2013). Positron emission tomography/computed tomography imaging in Merkel cell carcinoma: a study of 270 scans in 97 patients at the Dana-Farber/Brigham and Women’s Cancer Center. J Am Acad Dermatol.

[CR11] Sollini M, Taralli S, Milella M, Erba PA, Rubagotti S, Fraternali A (2016). Somatostatin receptor positron emission tomography/computed tomography imaging in Merkel cell carcinoma. J Eur Acad Dermatol Venereol.

[CR12] Taralli S, Sollini M, Milella M, Perotti G, Filice A, Menga M (2018). 18F-FDG and 68Ga-somatostatin analogs PET/CT in patients with Merkel cell carcinoma: a comparison study. EJNMMI Res.

